# Measuring Children’s Sodium and Potassium Intakes in NZ: A Pilot Study

**DOI:** 10.3390/nu10091198

**Published:** 2018-09-01

**Authors:** Helen Eyles, Neela Bhana, Sang Eun Lee, Carley Grimes, Rachael McLean, Caryl Nowson, Clare Wall

**Affiliations:** 1 Department of Epidemiology and Biostatistics, The University of Auckland, Auckland 1142, New Zealand; n.bhana@auckland.ac.nz (N.B.); slee925@aucklanduni.ac.nz (S.E.L.); 2 National Institute for Health Innovation, The University of Auckland, Auckland 1142, New Zealand; 3 Institute for Physical Activity and Nutrition, Deakin University, Geelong, VIC 3216, Australia; carley.grimes@deakin.edu.au (C.G.); caryl.nowson@deakin.edu.au (C.N.); 4 Department of Preventive and Social Medicine, University of Otago, Dunedin 9054, New Zealand; rachael.mclean@otago.ac.nz; 5 Department of Nutrition and Dietitics, University of Auckland, Auckland 1142, New Zealand; c.wall@auckland.ac.nz

**Keywords:** child, sodium, salt, potassium, New Zealand, Australasia

## Abstract

Low sodium and high potassium intakes in childhood protect against rises in blood pressure (BP) and risk of cardiovascular disease (CVD) later in life. Our aim was to pilot methods for collection of 24-h urine samples (gold standard) and diet recalls to assess sodium and potassium intakes and their food sources in 30 children aged 8–11 years at one New Zealand primary school. A diverse sample (*n* = 27) was recruited over a two-week period. All children provided a urine sample (71% complete) and interviewer-assisted 24-h diet recall (Intake24 software). Median (range) sodium intake was 2191 (1087 to 4786) mg/day (salt equivalent 5.5 g), potassium intake was 1776 (800–2981) mg/day, BP was 105 (84–129)/62 (53–89) mmHg, and sodium to potassium molar ratio was 2.0 (1.1–4.8). Frequent use of discretionary salt was uncommon. Major food sources of sodium were bread, pies and pastries, and bread and pasta-based dishes, and potassium were sauces and condiments, dairy products, and non-alcoholic beverages. Most participants provided adequate data and enjoyed taking part. A larger survey is warranted to confirm findings and inform a potential intervention(s). Small improvements to study procedures and resources should improve completeness of urine samples and quality of 24-h diet recall data.

## 1. Introduction

Dietary sodium and potassium are important determinants of blood pressure (BP) [1,2]. Diets high in sodium contribute 74,000 disability adjusted life years globally, mainly through their relationship with high BP and increased risk of cardiovascular disease (CVD) [3]. In New Zealand (NZ) and most other high-income countries, CVD is the leading cause of early health loss [4,5]. However, excess dietary sodium is also associated with increased risk of stomach cancer and kidney disease [6,7], and new research suggests there may be direct and indirect links with obesity [8,9]. Therefore, ensuring population sodium intakes are well within recommendations has important implications for public health.

In 2013, alongside several other countries, NZ committed to the World Health Organization (WHO) to reduce population salt intake by 30% towards 5 g (2000 mg of sodium) per day [10]. The dietary sodium intake of most adult populations is higher than this, including in NZ where those aged ≥15 years currently consume around 3300 mg per day [11].

In children, preference for salty food begins early in life [12], and there is strong evidence from meta-analyses of experimental and observational studies that reductions in children’s sodium intake lead to reductions in BP [13], and raised BP in childhood increases the risk of high BP later in life [1,14,15]. A lower sodium to potassium molar ratio in children also protects against rises in BP over a lifetime [16,17], and recent evidence suggests this measure may be superior and thus more politically relevant compared with separate sodium and potassium values for determining the relationships between BP and CVD [18] Therefore, the WHO strongly recommends a maximum level of 2000 mg of sodium and a minimum of 3510 mg of potassium per day for children adjusted downward based on the energy requirements of children relative to those of adults [19,20]; the recommended optimal sodium to potassium molar ratio for both adults and children is approximately one [19]. Children’s potassium intakes in NZ appeared adequate in the most recent (2002) Children’s Nutrition Survey [21], although these data are now out of date and nothing is known about the sodium intakes or sodium to potassium ratio of NZ children. In Australia where the food supply and children’s diets are similar to NZ, 72% of children aged four to 12 years exceeded the age-specific upper level (UL) for sodium intake and the sodium to potassium molar ratio was 2.4 [22].

One of the difficulties in collecting robust information on sodium and potassium intakes is that traditional dietary assessment methods such as food recalls and records are not considered adequate due to recall and social desirability bias, difficulty in accurately measuring salt added in cooking and at the table, and the need for brand-specific information on the sodium content of packaged foods. However, the majority of sodium (90 to 95%) and potassium (80 to 85%) excreted by the body is lost in the urine [23,24]. Therefore, a 24-h urine collection is the gold standard measure [23]. Twenty-four hour urine collections have been used successfully to assess the sodium and potassium intakes of large samples of children in the United Kingdom [25], Europe [26], and Australia [22]. The overall aim of this study was to pilot methods for collecting 24-h urine samples and diet recalls for assessment of sodium and potassium intake and their food sources in a sample of primary school children in NZ. Specific aims were to (1) evaluate children’s ability to self-collect 24-h urine samples by proportion of complete samples, (2) calculate daily intake of sodium and potassium and the sodium to potassium molar ratio, (3) identify major food sources of these electrolytes (4), explore discretionary salt use, (5) measure children’s BP, and (6) explore the feasibility and acceptability of collecting 24-h urine samples using a web-based dietary assessment tool (Intake24 [27]). The findings of this study will inform a larger survey of sodium and potassium intakes in an (ideally) representative sample of schoolchildren in NZ.

## 2. Materials and Methods

Study design: This was a cross-sectional pilot study conducted in one primary school in Auckland, NZ. Methods were based on those used in the successful SONIC (Salt and Other Nutrients in Children) study in Victoria, Australia [22,28].

Ethical approval and consent: Ethical approval was obtained from the University of Auckland Human Participants Ethics Committee (UAHPEC; 019774). Written informed consent was obtained from the participating Board of Trustees, Principal, teachers, students (assent), and parents/caregivers.

Outcome measures: Recruitment and retention rates, completeness of urinary sodium samples, average sodium and potassium intake and sodium to potassium molar ratio, major food sources of sodium and potassium, use of discretionary salt in cooking and added to meals, blood pressure (BP), and feasibility and acceptability of methods used to collect 24-h urine samples and diet recalls.

Participants, recruitment, and consent: Because this was a pilot study, a formal sample size calculation was not considered appropriate. Based on timeframe, capacity, and funding, our aim was to recruit 30 children, which based on estimated retention and collection of complete 24-h urine samples rates of 90% [28] would result in ~24 urine samples for analysis. Children aged 8 to 11 years (school years 3 to 6) who could speak and understand English were included, since Australian children in this age group were most likely to exceed sodium intake guidelines [29]. More than one child from the same family could take part.

We planned to recruit children from four classrooms at one diverse, co-educational primary school in the Auckland region. The 2017 Ministry of Education School Directory [30] was used to identify schools in deciles two to eight with a high proportion of Māori (indigenous New Zealanders) and Pacific. Schools were contacted one-by-one until a school agreed to take part. The first contact was via email to the Principal and Board of Trustees. The email included information about the study, i.e., study flyer, recruitment letter, and participant information sheet, and was followed up with a phone call one week later if no response was received. Once interest in participation had been confirmed, an informal meeting between the study Research Assistant and Principal was organised to answer any questions and discuss potential logistical issues. Briefing sessions were also held with the Board of Trustees and year 3 to 6 teachers to explain study procedures and provide an opportunity to tailor processes to best fit the school (see school checklist Supplementary Materials M1). Teacher consent forms were also signed at this meeting.

A combined briefing session was held with children and teachers in all participating classrooms, where study procedures were explained and any questions were answered. Recruitment envelopes containing an invitation letter, participant information sheet, and assent/consent forms were then sent home with all children. Information about the study was also included in the school newsletter and on the school Facebook page. Completed forms were returned to the classroom teacher or by freepost envelope. A study phone number was included on the participant information sheet and teachers were available to answer questions. Consenting children and parents/caregivers were assigned a unique, six-digit registration number in the on-line software used to securely store all study data (Research Electronic Data Capture; REDCap) [31]. To reimburse them for their time, the school was given a $NZ 500 sports voucher and participating students were provided with $NZ 20 movie vouchers.

Collection and assessment of outcome measures:

*Recruitment and retention rates* were calculated based on information from the study journal on the schools approached, and the number of individual participants who provided complete study information.

*Demographic information* was collected via an on-line survey in REDCap software including household income (voluntary), and child’s ethnicity (self-identified), age, gender, birth weight, existing medical conditions, and use of medication or dietary supplements. Parents and caregivers were sent an individualised link in an email and asked to complete the survey prior to collection of 24-h urines and dietary intake data. Daily reminder text messages and emails were sent to parents and caregivers who had not completed the survey within one week of the scheduled school visit.

*Twenty-four hour urine collections* were completed using standard procedures outlined by the WHO [32] on either a week or weekend day, as chosen by the child and their parents/caregivers. Children were issued with labelled urine collection kits and instructions at school following anthropometry and BP measures. Kits were provided in opaque blue carrier bags containing a 500 mL plastic jug to help with collection, a 2.5 L collection container, a funnel for transferring urine from the jug to the collection container, a ‘reminder’ safety pin for attaching to children’s underwear and hanger for the toilet door at home, and a sealable plastic bag of documents (instruction sheet, door hanger, safety pin, and collection record). Separate instructions were provided for children and adults (may be requested from corresponding author), and children’s instructions were discussed with them when the kits were issued. Briefly, children were instructed to empty their bladder, discard this urine, record the time as the start of collection, and collect all urine voided during the next 24 h. A second 2.5 L container was provided to children who elected to collect on a school day so that they could leave partial samples at school. School samples were collected in a separate, secure toilet in the school sick bay where children could leave partial samples during the day. The hours of collection and any missed collections or spillages were recorded on a form which was returned with the full urine sample to school. Twenty-four hour urine samples were collected from the school by the study Research Assistant. The total volume was recorded and 2 × 10 mL aliquots were prepared and stored at −4 °C in preparation for analysis. Samples were analysed at an accredited laboratory (University of Otago laboratories) [24]. Urinary sodium and potassium were analysed using a Hitachi Cobas C311 analyser, using an Ion selective electrode. Urinary creatinine was analysed on the Cobas C311 using a colorimetric assay based on the Jaffe method [33]. If the sample collection time was not exactly 24 h but within 20 to 28 h, urinary sodium, potassium and creatinine were standardized to a 24-h period. Children with SBP and DBP measures >90th percentile for their age, gender and height were sent a message home suggesting they may wish to follow up this result with their general practitioner.

*Completeness of 24-h urine samples* was assessed by total volume >300 mL, collection time >20 h and <28 h, the participant reported one or fewer missed collections, and urinary creatinine excretion >0.1 mmol/kg body weight per day.

*Sodium and potassium intakes* were calculated using gold standard 24-h urine collection data as median (range), due to the small sample size and skewness of the data. Medians were calculated for all urine samples and for complete samples separately.

*The sodium to potassium molar ratio* was calculated by converting daily amounts of sodium and potassium consumed from the urine collection data (mg) to mmol of intake using the molecular weights of sodium and potassium (23 and 39.1, respectively).

*Twenty-four hour diet recalls* were collected by a trained Research Assistant during or after school, the day following urine collection, using a NZ version of the on-line interactive Intake24 software [27]. Intake24 employs standard multiple pass dietary recall methods [34], includes predominantly generic food composition data from the 2014 Concise NZ Food Composition Tables [35], and has been validated for use as a self-administered tool in 11 to 24 year olds [36]. However, as this was the first time the software had been used by younger children, diet recall interviews were completed face-to-face together with the participating child and where possible, their parent(s)/caregiver(s). Participants were given the option of entering their own data with help from the Research Assistant, or for the Research Assistant to enter the data for them. Participants were asked if they added salt to each food or recipe. Intake24 allows for a minimum of 0.25 teaspoons (tsp) of salt to be added. Therefore, discretionary salt was added in 0.25 tsp increments and 0.25 tsp was added as the standard for a ‘shake’ of salt. Generic information on the sodium content of all packaged foods was replaced with brand-specific information from the NZ Nutritrack database [37], a national database comprising annually updated information on the composition of packaged foods sold at major NZ supermarket products. The generic sodium content from the Intake24 software database was used for all fresh foods and any foods missing from Nutritrack.

*Major food sources of sodium and potassium* were assessed using 24-h recall data.

*Discretionary salt use* was assessed in the on-line REDCap survey completed by parents at the time of collection of demographic information. Three questions on discretionary salt use were included from the Australian SONIC study [28]: (1) Do you usually add salt during cooking? (2) Do you usually place a salt shaker on your table at mealtimes? and (3) Does your child usually add salt to their meal at the table or in food preparation? Participants were given four options to answer each question: Yes, Yes sometimes, No, or Don’t know.

*Blood pressure and anthropometric measures* were collected using standard WHO procedures [32] by a trained Research Assistant during school hours, prior to collection of dietary data and urine. Weight was measured to the nearest 0.1 kg on a flat surface using a calibrated electronic scale. Children were asked to remove shoes and socks. Height was measured to the nearest 1 mm on a calibrated laser stadiometer on a flat, hard floor. Waist circumference was measured to the nearest 1 mm over light clothing using a constant tension tape measure. Blood pressure was measured on the right arm using an OMRON automatic BP monitor in a seated position after 10 min rest, on the day of urine collection. The average of two measures was recorded for weight, height, and waist circumference unless the measurements were >0.1 kg, 0.5 cm, or 1.0 cm respectively, in which case the average of three measures was recorded. The average of three measures was recorded for BP. Children’s BP measures were compared with gender, age, and height percentiles from the American Academy of Pediatrics [38]. The percentage of children with ‘prehypertension’ was not calculated because the average of measures on three separate occasions is required for this definition.

*Feasibility and acceptability of methods used to collect urine samples and 24-h dietary recall data* were assessed in face-to-face interviews with children and their parents/caregivers at school, prior to diet recall interviews (for urine samples) and following diet recall interviews (for 24-h recall data). Interviews followed a semi-structured format (Supplementary Materials M2) and responses were recorded verbatim into an Excel spreadsheet. Teacher responses were collected via an on-line REDCap survey [31]. Responses from all groups were combined in Excel and analysed using inductive thematic analyses.

## 3. Results

### 3.1. Recruitment and Retention

Recruitment of the school and participating children occurred over a six-week period from August to October 2017. Three primary schools were approached with the third school agreeing to take part. Board of trustee and teacher consents were obtained over three weeks with all six teachers from year 3 to 6 classrooms agreeing to take part. Twenty-nine assent and consent forms were returned over a two-week period from 118 children (25% response rate). Two children dropped out before providing any demographic information, one of which did not respond to the parent/caregiver survey and the other was withdrawn by the school who determined they could not fully understand and speak written English (93% conversion rate).

### 3.2. Demographics of Particpating Children

Demographics of the 27 participating children are shown in Table 1. Approximately half were girls (*n* = 13), who were slightly older and heavier than boys. The most common ethnicity children identified with was Pacific Island (*n* = 31) followed by NZ European (*n* = 10) and Māori (*n* = 10). More than half of the children (19/27) were living in a household with a total income lower than (<$70,000) the national median (~$80,000 [39]).

### 3.3. Completeness of Urine Samples

Eight of 27 (29%) urine samples were considered incomplete due to the duration of collection being outside 20 to 28 h (*n* = 3), total volume <300 mL (*n* = 3), more than one collection reported as missing (*n* = 3), or urinary creatinine excretion <0.1 mmol/kg per day (*n* = 6; some samples failed multiple criteria for completeness).

### 3.4. Sodium and Potassium Intake and Sodium:Potassium Molar Ratio

The median (range) sodium intake of children as assessed by complete 24-h urine samples (*n* = 19) was 2191 (1087 to 4786) mg per day (salt equivalent 5.5 g/day), slightly higher than for all samples (*n* = 27; 1943 (831 to 4786) mg per day (salt equivalent 4.8 g/day; Table 2). Corresponding values for potassium intake were 1776 (800 to 2981) and 1696 (434–2981) mg per day. Median sodium intake was higher for boys than girls. In contrast, boys had a slightly lower median potassium intake despite also having a lower energy intake than girls (Supplementary Table S1). Using the complete urine data, 10 of 19 children (53%) consumed more sodium than the current UL for NZ children aged 9 to 13 years (2000 mg/day or 5 g salt [40]), which aligns with the WHO salt recommendation for adults [19]. All 19 children consumed less potassium than the current Adequate Intake (AI) for NZ boys (3000 mg/day) and girls (2500 mg/day) aged 9 to 13 years [41]. The median sodium to potassium molar ratio was much higher than the WHO optimal value of one [19] 2.0 (1.1 to 4.8), and was higher for boys than girls (Table 2). All 19 children with complete urine samples had a sodium to potassium molar ratio >1.0.

### 3.5. Major Food Sources of Sodium and Potassium

**Figure 1:**
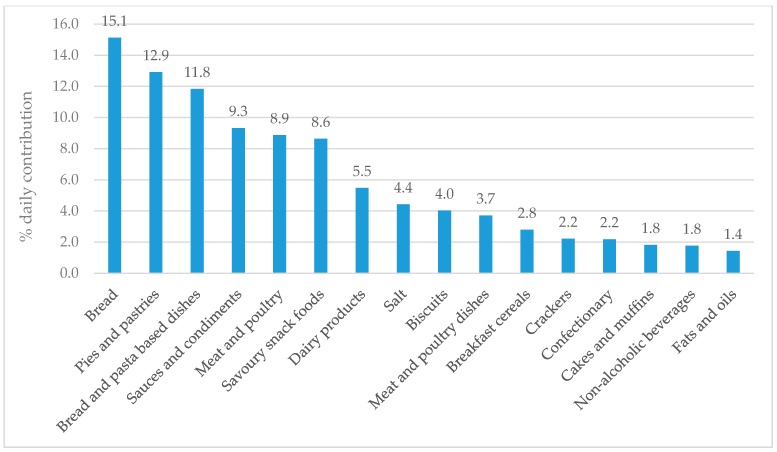
Daily contribution of sodium from 16 major food groups (≥1% daily contribution) among participating children (*n* = 26).

**Figure 2:**
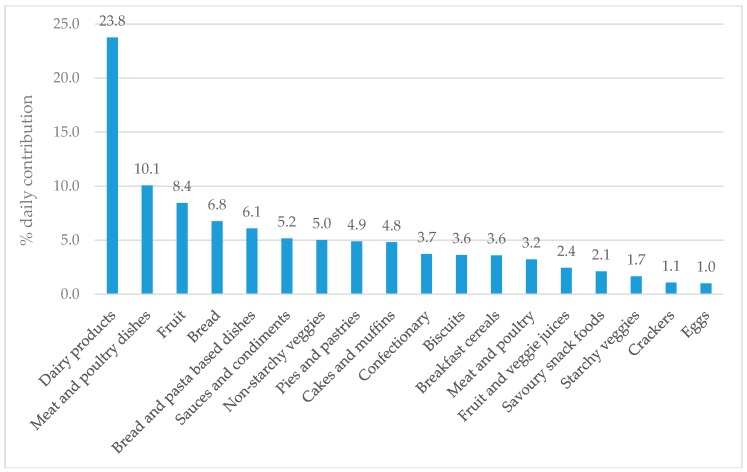
Daily contribution of potassium from 17 major food groups (≥1% daily contribution) among participating children (*n* = 26).

Twenty-two children (81%) had a parent or caregiver present during collection of their 24-h dietary recall. The overall nutrient content of participant’s diets is available in Supplementary Table S1. One diet recall was removed from the analysis as an outlier for potassium intake (6.6 mg per day; >2SD’s below the mean (2081 mg per day). Figure 1 and Figure 2 show the major food groups contributing ≥1% of daily sodium and potassium intake, respectively. The top contributor to daily sodium intake was bread (15.1% daily) followed by pies and pastries, bread and pasta dishes, sauces and condiments, meat and poultry, savoury snack foods and dairy products, all of which contributed ≥5%. Salt added directly to food contributed 4.4% to daily intake. The top contributor to daily potassium intake was dairy products (23.1%) followed by meat and poultry dishes, fruit, bread, bread and pasta dishes, sauces and condiments, and non-starchy veggies (all ≥5%).

### 3.6. Use of Discretionary Salt

Eleven of 27 parents and caregivers (41%) reported adding salt to food they prepare for their children either always or often. Only two parents/caregivers reported never adding salt to food they prepare for their children. In contrast, no parents or caregivers reported that their children always or often add salt to their food at the table, and 11 reported that their children never add salt at the table. Five (19%) parents/caregivers reported always or often putting a salt shaker on the table during mealtimes, and 10 (37%) reported never placing a salt shaker on the table. Eleven parents/caregivers reported that they were trying to cut down on the amount of salt they eat.

### 3.7. Blood Pressure

The median (range) systolic blood pressure (SBP) of all children (*n* = 27) was 105 (84 to 129) mmHg and diastolic blood pressure (DBP) was 62 (53 to 89) mmHg. Corresponding values for boys (*n* = 14) were 105 (84 to 129) mmHg and 63 (54 to 89) mmHg, and girls were 107 (92 to 119) mmHg and 62 (53 to 79) mmHg. Twenty two children (82%; 12/13 girls and 10/14 boys) had a SBP <90th percentile, and 22 (82%; 11 girls and 12 boys) had a SBP <95th percentile for their gender, age, and height [38]. Twenty four children (89%; 11 girls and 13 boys) had a DBP <90th percentile, and 24 (98%; 11 girls and 13 boys) had a DBP <95th percentile.

### 3.8. Feasiblity and Acceptibility of Methods for Collecting 24-h Urine Samples and Diet Recalls

#### 3.8.1. 24-h Urine Samples

Most children (17/27) elected to collect their urine sample on a week/school day. Inductive thematic analysis of parent/caregiver and teacher interviews elicited four key themes for feasibility (participants satisfaction, autonomy/supervision, student learning experience, and ease of collection), and three for acceptability (communication, instructions, and equipment) of procedures used to collect urine samples. Cultural aspects were also identified. Themes, explanations and supporting quotes are summarised in Table 3. Overall, most children could self-manage collection of their own urine, enjoyed taking part in the study, and said they would take part again. Teachers thought the study aligned well with topics such as wellbeing, family connection, and science currently taught, and study procedures caused little disruption in the classroom. However, there were mixed feelings regarding how satisfied students, parents and caregivers felt taking part, and a need was identified to communicate reminders to parents/caregivers via text message rather than email. Further, it was not clear to parents/caregivers that the urine record sheet needed to be returned to study researchers and the reminder door hanger and safety pin were unused by many children. Some children also found they were shy about discussing collection of their urine, and there was some discomfort associated in doing so. There was also a need to acknowledge cultural safety of urine storage in the instructions (Table 3).

#### 3.8.2. 24-h Diet Recalls

All diet recalls were completed over the same 24-h period as urine collections. Diet recalls took on average 33.8 (16.7) min to complete (range, 15 to 26 min). Of the 428 individual foods and beverages consumed by participating children, 233 (54%) had their sodium data replaced with brand-specific values from the 2017 Nutritrack database [37], and the remaining foods had a generic sodium value from the Intake24 food composition database [35]. All 27 children and their parents/caregivers reported that Intake24 helped them to remember and record the foods and beverages consumed over the past day. Only two participants reported difficulty in using the programme, one who felt ‘it was confusing because there were too many things to do’ and another who ‘..didn’t want to type because he was afraid of making spelling mistakes’. All parents and children felt the face-to-face instructions were helpful and couldn’t think of anything that could be done to improve them e.g., ‘Good to have xxx present to help/present’. Four parents/caregivers made suggestions to help better collect children’s food and beverages intakes in the future. These were to collect information away from special holidays such as Halloween, provide fewer choices in the software, provide a chart or diary for writing information down as it was consumed (like a food record), and be aware that children might not understand the different brands for packaged foods.

## 4. Discussion

We successfully recruited and retained a diverse sample of schoolchildren (*n* = 27) aged eight to 11 years from one NZ primary school, and during one term were able to collect 24-h urine samples and diet recalls for assessment of sodium and potassium intake. The majority (70%) of children provided complete urine samples. However, because our study was a pilot involving a small number of children, we could not accurately calculate population estimates of children’s sodium and potassium intakes, or BP. Nonetheless, we found ~50% of children consumed more sodium than national [40] and WHO recommendations [19], no children consumed adequate potassium, and only one child had a sodium to potassium molar ratio in the healthy range (≤1.0). Boys had a higher sodium to potassium molar ratio compared with girls. The majority of children (>80%) had singular SBP and DBP measurements within the 90th percentile for their age, sex, and height. The major food sources of sodium in our sample of children were bread, pies and pastries, and bread and pasta dishes (all ≥10% of total sodium intake), and the major food source of potassium was dairy (23.1%; all other foods contributed <10%). Less than half of parents and caregivers added salt to food prepared for children, and less than half of children added salt at the table.

Overall, most children, teachers, parents and caregivers understood the study requirements, found procedures easy to follow, noticed little disruption to the classroom, could identify with the study given it aligned well with topics already taught in class, and enjoyed taking part. Importantly, children were able to self-manage collection of their own urine, and with help from the study researcher and parents/caregivers, most could remember their food and beverage intake from the previous day and use Intake24 software to record it. Feedback from participants was useful to identify the following improvements to procedures: aim to improve student satisfaction, communicate with parents using text rather than email, be clear about the need to complete and return the urine record sheet, address how children feel about discussing collection of their urine, acknowledge cultural-specific notions of safety in urine collection instructions, and explain food brands to children when collecting dietary intake information.

There were several strengths to our pilot study, including that methods were based on standard WHO procedures previously used to successfully collect 24-h urines and diet recalls from children of a similar age in Australia [28]. We also managed to recruit, retain and collect data for a diverse sample of schoolchildren within a short (two-week) time period. Successful recruitment and retention indicates the feasibility of completing a larger survey, and the diversity is important given high BP and CVD are present in unequally high proportions in low income, Māori and Pacific peoples in NZ [43].

However, there were also some study limitations, such as the fact that BP was only measured once, and the average of measures on three separate occasions is required to define a child as having high BP [38]. Nonetheless, our findings that ~20% of children had a SBP >90th percentile and 10% had a DBP >90th percentile on one occasion for their age, gender, and height are still a useful indication of the BP status of the sample. Other limitations of our study include that the minimum amount of added salt in Intake24 was 0.25 tsp, there was a limited depth of responses from parents, caregivers, and children regarding the feasibility and acceptability of Intake24, and we had a low recruitment rate within participating classrooms (25%) and high proportion of incomplete samples (30%). The minimum value of 0.25 tsp of salt may have artificially inflated the mean daily salt intake and affected the order of major food sources of sodium in children’s diets. However, only two participants reported adding a ‘shake’ of salt with the remainder reporting ≥0.25 tsp as part of a recipe, and thus the impact on study findings would have been minor. Recruitment rates of previous urinary excretion studies in British and Portuguese children of a similar age were higher than for our pilot (52% (169/324 8 to 9 year olds and 41% (202/488) [44], respectively), although our recruitment rate should still be considered reasonable as it was higher than that of the recent Australian SONIC study (5%; 4 to 12 years; 780/14,509) [22]. The proportion of children with complete samples observed in our pilot (70%) was also lower than larger international studies using the same criteria (79% in South London [25] and 89% in Australia [22]). Nonetheless, improving the instructions for urine collection and use of the urine collection sheet should lead to a higher proportion of complete samples in any future study involving NZ children. Improved instructions may also increase the level of agreement between sodium and potassium intakes from urines and diet recalls, which was poor for our sample (Supplementary Figure S1A–D). However, comparing these two methods for assessment of sodium and potassium was not an objective of our pilot.

Despite the small sample size of our study, it is still useful to compare findings with larger international electrolyte excretion studies to provide a context for NZ children. The median daily salt intake of eight to 11 year old children in our pilot (*n* = 19) was 5.5 g (2191 mg sodium), slightly higher than for children of a similar age in Britain [25] and Germany [26], but lower than comparators in Australia [22] and Spain [45] (Table 4). Fewer data are available on children’s 24-h potassium excretion, but in our sample median daily intake (1776 mg) was slightly lower than for similar aged children in Australia [22] and Spain [45]. Similar to our sample salt excretion of 7 to 12 year old children internationally is higher for boys than girls, as would be expected due to their higher energy intakes (Table 4). However, boys in our study had a slightly lower potassium intake than girls, whereas the potassium intake of boys in most other countries was higher than for girls. This could be partly explained by the fact that girls in our sample were slightly older and heavier than boys.

No comparable 24-h urinary sodium or potassium excretion data currently exist for NZ children. However, the sodium to potassium molar ratio from urinary excretion data for NZ adults (aged 18 to 64 years; *n* = 299), was much lower than in our sample of children (1.32 vs. 2.0, respectively) [11]. The sodium to potassium molar ratio is relatively insensitive to completeness of the urine collection, thus suggesting our findings are robust. The most recent dietary intake data (2015) for NZ children are from a cohort study, the Auckland Birthright Collaborative (ABC), where 24-h assisted food records were collected in children (appropriate size for gestational age) at seven years (*n* = 564) and 11 years (*n* = 609). ABC study findings are comparable to our pilot for salt (5.4 and 5.6 g per day, respectively, vs. 5.5 g per day), but lower (in our sample) for potassium (2416 and 2578 mg per day, respectively, vs. 1776 mg per day) [46]. Children in our pilot study consumed a median (range) 113 (0 to 694) g of fruit and vegetables (including juice) per day, much lower than the guideline of 5+ serves (~400 g) per day for NZ children and young people [47], which may at least partially account for their low potassium intake. Previous national surveys illustrate the wider population of both NZ and Australian children consume far fewer servings of fruits and vegetables than recommendations [43,48].

In our pilot study, the top five food sources of sodium for children were bread, pies and pastries, bread and pasta-based dishes, sauces and condiments, and meat and poultry, similar to those identified for NZ adults both in terms of individual intakes (bread, processed meat and sausages, sauces, potatoes and kumara, and breakfast cereals [50]) and purchases (bread, cheese, butter and margarine, milk, bacon, and table sauces) [51]. Pilot study children also had similar foods sources of sodium to Australian children in the SONIC study (cereal and cereal products; cereal and cereal product dishes; meat, poultry and game products and dishes; milk products and dishes; and savoury sauces and condiments) [22], who share a similar dietary pattern and food supply in NZ children. Major food sources of potassium in NZ children were also similar between these three groups, with the exception of vegetables which were not a top three contributor in our study (dairy products, meat and poultry dishes, and fruit), but were for adults in the 2008/09 NZ Adult Nutrition Survey (potatoes kumara and taro, other vegetables, non-alcoholic beverages, fruit and milk) [52]; and Australian children in SONIC (vegetable products and dishes, fruit products and dishes, and cereal and cereal products) [22].

Approximately half of the children with complete urine samples in our sample exceeded the NZ UL and WHO recommendation for salt intake (5 g per day), and all children consumed less potassium than the NZ AI and had a sodium to potassium molar ratio of >1.0. These findings suggest that the sodium and potassium intakes of the wider population of eight to 11 year olds in NZ are unlikely to be ideal, and a larger more representative survey is warranted to confirm findings and improve accuracy of measures. The most recent NZ Children’s Nutrition Survey was completed in 2002 [21], and although there appears to be increased interest from the NZ Government, there are no current plans to update this survey in the near future. Furthermore, any such national survey would be unlikely to include 24-h urine samples due to the cost and participant burden. Therefore, a smaller survey in a diverse but focused age group of NZ children may be more feasible. Assuming an SD of 1000 mg per day in sodium intake from the current study and the previous Australian SONIC study [22], we estimate a sample of 400 children aged eight to 11 years would give an estimated mean sodium intake (mg per day) with a margin of error of 100 mg.

Any future, larger survey with NZ children should also take into account the learnings from this pilot, including: (1) the need for flexible study methods adaptable to individual school communities, (2) the importance of providing parents with a choice between online or paper surveys, (3) the need to review and amend urine collection instructions with parents and children, (4) the usefulness of text reminders for parents, and (5) the need to support parents to complete 24-h recalls in a flexible time slot to reduce study time and participant burden.

## 5. Conclusions

In conclusion, we have shown that it is possible to collect 24-h urine samples and diet recalls from eight- to 11-year-old children in a NZ school setting. Most children, teachers, parents and caregivers provided adequate data for analysis, enjoyed taking part, and said they would do it again. With small improvements to study procedures and resources it should be possible to increase the proportion of complete 24-h urine collections and improve the ability of children to recall and record their previous days dietary intake using Intake24 software. Our findings suggest that the sodium and potassium intakes of the wider population of NZ children are unlikely to be ideal, and thus a larger more representative survey is warranted. Any future survey should aim for a diverse population, take into account learnings from the current pilot study, and for feasibility purposes may need to be focused on a specific age group. Nonetheless, such a survey is important, not only to confirm the findings of the current pilot, but also to inform an intervention(s) to reduce sodium intakes and increase potassium intakes in NZ school children.

## Figures and Tables

**Table 1:** Demographics of the 27 children who participated in the study.

Demographic (Mean, SD)	Girls (*n* = 13)	Boys (*n* = 14)	Total (*n* = 27)
**Age (years) ^1^**	9.1 (8, 11)	8.6 (8, 10)	8.9 (8, 11)
**Weight (kg)**	46.9 (10.3)	38.8 (11.9)	42.7 (11.7)
**Height (cm)**	143.9 (7.8)	138.1 (6.4)	140.9 (7.5)
**Waist circumference (cm)**	75.7 (9.8)	68.1 (12.0)	71.8 (11.5)
**Ethnicity ^2^ (*n*)**			
European	4	6	10
Māori ^3^	3	7	10
Samoan	6	4	10
Cook Island Māori	3	0	3
Tongan	7	8	15
Other ethnicity ^4^	1	5	6
**Household income (n)**			
<$NZ 70,000	11	8	19
>70,001	2	3	5
Declined to answer	0	3	3

^1^ mean (range), ^2^ Participants could identify with more than one ethnicity, ^3^ Indigenous New Zealanders, ^4^ Including other Pacific Island groups.

**Table 2:** Twenty four hour urinary electrolyte excretion overall and by sex.

Median (Range)	Girls	Boys	Total
**24-h urine (all samples; *n*)**	**13**	**14**	**27**
Sodium (mg/day)	1494 (870–2992)	2504 (831,4786)	1934 (831–4786)
Salt equivalent (g/day)	3.7 (2.2–7.5)	6.3 (2.1–12.0)	4.8 (2.1–12.0)
Potassium (mg/day)	1738 (434–2244)	1589 (495–2981)	1696 (434–2981)
Sodium: potassium (molar ratio)	1.7 (1.1–6.2)	2.9 (1.4–7.2)	2.5 (1.1–7.2)
Volume output (mL/day)	526 (127–897)	644 (202–1167)	762 (45–1167)
**24-h urine (complete only; *n*)**	**9**	**10**	**19**
Sodium (mg/day)	1623 (1087–2992)	2407 (1381–4786)	2191 (1087–4786)
Salt equivalent (g/day)	4.1 (2.7–7.5)	6.0 (3.5–12.0)	5.5 (2.7–12.0)
Potassium (mg/day)	1845 (1504–2244)	1659 (800–2981)	1776 (800–2981)
Sodium: potassium (molar ratio)	1.6 (1.1–2.6)	2.9 (1.4–4.8)	2.0 (1.1–4.8)
Volume output (mL/day)	767 (468–897)	703 (450–1167)	762 (450–1167)

**Table 3:** Themes elicited from interviews with parents/caregivers (*n* = 27) and teachers (*n* = 6) on the feasibility and acceptability of 24-h urine collection.

Theme	Explanation	Supporting Quotes
Feasibility		
Participant satisfaction	Most children said they would take part in the study again. However, there were mixed feelings regarding how satisfied children felt taking part.	*“It was weird to collect.. there was nothing I didn’t like. I would take part again” (Child)* *“Easy to do, but I didn’t really like it. Would do it again” (Child)*
Autonomy/supervision	Most children were able to self-manage collection of their own urine. Some parents chose to start their child on a weekend day so they could monitor the collection process. The age group was deemed appropriate with children having a sense of responsibility and autonomy over their own collection.	*“It was all straight forward and easy to manage. The children did it themselves, didn’t need to do much. Classroom interference was barely noticeable” (Teacher)* *“Better to do it on the weekend at home. Able to watch and make sure it was collected. Tricky to do at school” (Parent/caregiver)*
Student learning experience	Children said given the opportunity most children would take part in the study again because it was fun, interesting, and they were able to learn new things. The study support topics hauora (well-being) and whanaungatanga (relationship, sense of family connection) taught earlier in the school year and developed their interests further. The school and participating students were appreciative of being given the opportunity to take part. Some children commented that taking part meant they were helping with something bigger, demonstrating the vision, values and competencies of the NZ school curriculum.	*“The students were really excited to participate in the science study. We do a lot of science in the classroom so they were eager to get involved” (Teacher)* *“Would do it again because it’s about how much salt we’re eating. It was weird because we had to catch our pee and hold the jug close to us” (Child)* *“Best part was doing the blood pressure and stuff”*
Ease of collection	Overall, the children found it easy to collect their urine.	*“Collected it all. Just a few drops spilled. Easy to do” (Child)* *“Collected full time. No spills. No problem collecting” (Child)*
**Acceptability**		
Communication	Text reminders were the preferred method of contact. The email with the on-line survey had a low initial response rate despite several reminder emails.	*“Survey with consent form. Text reminder helped—prefer text instead of email” (Parent/caregiver)* *“Text reminders were helpful. Would have forgotten otherwise” (Parent/caregiver)*
Instructions	Overall, families and teachers were positive about the study, felt well-informed, and understood the requirements. However, there was disconnect observed between these comments and the correct return of the record sheet (for urine collection timeframe and spillages) and collection bottle. Only six participants initially returned the record sheet, and 10 returned the collection bottle with all of the correct documentation.	*“Instructions were all good. Instructions needed more pictures” (Parent/caregiver)* *“Instructions easy to relay to the children. Didn’t read the children’s instruction sheet Mum showed us how to do it” (Child)*
Equipment	The jug and funnel were considered key components of the collection kit, but some children would have liked a larger jug to catch their urine and avoid chance of contact. Few children used the reminder door hanger and/or safety pin. Many kits were returned with the instructions and record sheet unopened in the resealable bag.	*“Equipment was good. Funnel and jug made it easy to do” (Child)* *“Big jug so it doesn’t splatter when it gets filled to the top” (Child)* *“He wasn’t interested in the door hanger. Kept the bottle in the bathroom as a visual reminder” (Parent)*
**Cultural aspects**	Most families were accepting of the methods required and few reported any cultural concerns pertaining to study methods. One parent shared a concern around storage of urine containers in a bathroom—because other small children were in the house the containers were stored above the child’s head which was considered tapu (sacred, restricted) ^1^. Many children were shy of talking about their experiences (whakamā) and appeared embarrassed to talk about going to the toilet or collecting their urine. Comments expressed some discomfort/anxiety around touching their urine and parts of their body that are not typically spoken about.	*“No cultural issues, no I didn’t have concerns about cultural appropriateness” (Parent/caregiver)* *“Giving each student the blue bag with all the utensils was awesome. They were able to carry it home/back to school with ease, and although everyone else knew what was inside it there was no mocking or anything at all (Parent/caregiver)* *“..placed the bottle on the window sill, which is above where you would stand, so tapu, be good to acknowledge this in instructions” (Parent)*

^1^ [42].

**Table 4:** Previous studies examining twenty-four hour urinary electrolyte excretion overall and by sex in children aged approximately seven to 11 years.

Country	Year	Age (Years)	*n*	Salt (g)			Potassium (mg)		
				Girls	Boys	Total	Girls	Boys	Total
Australia [22]	2017	9–12	383	6.1	7.0	6.6	1838	2111	1994
Britain [25]	2014	8–9	111	4.8	4.7	3.8	NR	NR	NR
Germany [26]	2011	7–10	83	NR	NR	4.6	NR	NR	NR
Italy [49]	2015	8–10 (G) & 9–11 (B)	408	5.6	7.8	NR	1290	1642	NR
Portugal [44]	2015	8–10	163	6.0	7.3	NR	1682	1701	NR
Spain [45]	2017	7–11	205	7.0	8.2	7.8	1826	2021	1932

NR = not reported G = girls, B = boys.

## Supplementary Materials

The following are available online at http://www.mdpi.com/2072-6643/10/9/1198/s1, Materials M1: School checklist, Materials M2: Semi-structured interview follow-up questions, Table S1: Energy and nutrient composition of children’s diets from 24-h recalls, Figure S1A–D: Scatter plots and Bland Altman plots comparing sodium and potassium intakes as assessed by 24-h urine samples and diet recalls.
