# Supplementary material for: Measuring Children’s Sodium and Potassium Intakes in NZ: A Pilot Study
Source: Nutrients. 2018 Sep 1;10(9):1198. doi: 10.3390/nu10091198 (PMC6164957; doi:10.3390/nu10091198)
Supplement: Supplementary file 1 [file nutrients-10-01198-s001.zip › Supplementary Materials_Submitted.docx]

Supplementary materials M1: School Checklist

| **School:** |  |
| --- | --- |
| **Principal:** |  |
| **Alternative contact(s) name:** |  |
| **Main contact phone:** |  |
| **Main contact email:** |  |

# Key Milestones

| **Activity** | **Date** |
| --- | --- |
| **Briefing with Board of Trustees** |  |
| **Board of Trustees consent received** |  |
| **Principal consent received** |  |
| **Planning meeting with Principal** |  |
| **Teacher briefing session** |  |
| **Teacher consent received** |  |
| **Participant information packs distributed to students** |  |
| **Consent forms returned** |  |
| **Online survey emailed to parents** |  |
| **Baseline measurements/Urine collection kits issued** |  |
| **24-hour urine collection start** |  |
| **24-hour diet recall with parents/caregivers** |  |
| **Follow-up interviews with teachers** |  |
| **Science lessons** |  |

# Classroom List

| Target student | | Year 3 to 6 (aged 8-11years) | |
| --- | --- | --- | --- |
| Target number of participants | | 30 students (max 35) | |
| **Year** | **Room #** | **Roll per class** | **Teacher** |
| **Year 3** |  |  |  |
|  |  |  |  |
| **Year 4** |  |  |  |
|  |  |  |  |
|  |  |  |  |
| **Year 5** |  |  |  |
|  |  |  |  |
| **Year 6** |  |  |  |
|  |  |  |  |

| How will consent forms be returned? i.e.:   - class teacher and email sent to RA - freepost back to RA |  |
| --- | --- |
| Additional communications required for school? i.e.:   - notice in school newsletter - info session for parents/caregivers |  |
| Date next newsletter is to be issued |  |
| Newsletter extract to be emailed to: |  |
|  |  |
|  |  |

# Data Collection

| **Visit 1: Body measurements and blood pressure**   - Body measurements to be taken: height, weight, waist & hip circumference, blood pressure - Completed during school hours - 30 minutes per child - Child will be issued with their urine collection kit at this visit and taken through instructions of how to complete their collection. - Parents/caregivers will be contacted following this to schedule their 24-diet recall interview and confirm the day their child will be collecting their sample. | |
| --- | --- |
| - Where can we complete measurements? |  |
| - Can participants assist with being “runner” i.e telling next student when it is their turn |  |
| Requirements:   - Private, quiet room - 3 chairs - 1 table |  |

| **Visit 2: Diet recall interview**   - Interviews will be facilitated by Masters Student - To be completed with parent/caregiver present, therefore after school appointments - 30-45min per interview | |
| --- | --- |
| - Is there a room available after school hours to use for interviews? |  |
| - What time does school finish? (when can we start interviews) |  |
| - What time can we use the facilities/stay on school site till (i.e. what time would we need to finish interviewing by?) |  |
| - Will a staff member/caretaker be present (locking up etc) |  |
| Requirements:   - Private, quiet room - 4 chairs - 1 table - Wireless internet connectivity - Area for other families to wait |  |

| **Follow-up interviews with teachers**   - Final interview with teacher to ask about their experience at end of study - No more than 10 minutes - Can be completed individually or as a group discussion (15-20min to gather all feedback) | |
| --- | --- |
| - Best time to meet with teacher(s)?   Before school/ morning tea/ lunch/ after school |  |

| **Urine Collection:**   - Children will have the choice to complete a weekend (at home) or weekday (at school) collection. - This will be confirmed when RA contacts the family after child has been issued their kit. - Because we are making appointments with families to complete their diet recall interview, and there are only limited after-school appointments available each day, not all children will be collect their urine on the same day. There will only be approx. 4-6 children collecting their urine at school per day. - RA will collect full bottles from school, approx. 9.30am each day | |
| --- | --- |
| Are there provisions for a “participant’s bathroom”? |  |
| Secure area for bottles to be stored during school-day collection?  Will it be safe for bottles to be stored in participant bathroom? |  |
| Where will full bottles (that are ready for collection) be stored? |  |
| What are the schools clean up procedures/protocols in case of spillages at school |  |
| What support services are available to assist children who may be feeling uncomfortable/ bullied for taking part?   - Public Health Nurse (PHN)? - Social Workers in Schools (SWiS)? |  |

| **Information for Research team while on site** | |
| --- | --- |
| Is there internet available which we may access while on site for the diet recall interviews? |  |
| Any health and safety concerns the research team needs to be aware of when working on site during and after school hours? |  |

**Any other comments/suggestions/considerations to be accounted for?**

**Supplementary Materials M2: Semi-structured interview follow-up questions**

**Semi-structured interview questions for follow-up interviews with parents/caregiver**

1. Did you and your child feel comfortable participating in the study? Why? Why not?
2. Was your child able collect a *complete* 24-hour urine sample?
3. Can you please tell me if there was anything you or your child found difficult about collecting the urine samples?
4. Can you please tell me if there was anything you or your child found easy about collecting the urine samples?
5. Can you please tell me if there was anything you or your child found difficult about collecting the diet recalls?
6. Can you please tell me if there was anything you or your child found easy about collecting the diet recalls?
7. Can you please tell me what you thought about the instructions you were given?
   I.e. clear/useful/informative/easy to understand/confusing/lacked detail etc.
8. Do you think there was any information that was missing/could have been useful for collect the 24-hour urine samples?
9. How well did your child understand the instructions?
10. Do you think there were any differences in the information provided to you and what was provided to your child?
11. (IF PARTICIPANT WITHDREW FROM STUDY) Can you please tell me the main reason for withdrawing from the study?
12. We would like to gather some further information about your thoughts regarding sodium intake and health. Could you please indicate on a scale of 1-5 how much you agree or disagree with the statement “In the long term, eating too much salt during childhood may have harmful effects on children’s health”. (1= strongly disagree, disagree ,neither agree nor disagree, agree or 5=strongly agree)
13. Medical research suggests that children eat more salt than is good for them. On a scale of 1-5, do you think more action needs to be taken to reduce the salt in foods targeted at children? (1= strongly disagree, disagree ,neither agree nor disagree, agree or 5=strongly agree)
14. Please share any additional comments/feedback you may have about the study overall, especially that could help us to improve our methods next time?

**Semi-structured interview questions for follow-up interviews with teachers**

1. Did you feel comfortable participating in the study?
2. Did the students discuss the study inside/outside of class-time, other than when they were briefed about the study?
3. Can you recall any of the comments that were shared?
4. Can you please tell me if there was anything you found difficult about collecting the urine samples?
5. Can you please tell me if there was anything you found easy about collecting the urine samples?
6. Can you please tell me what you thought about the instructions you were given?
   I.e. clear/useful/informative/easy to understand/confusing/lacked detail etc.
7. Do you think there was any information that was missing/could have been useful for collect the 24-hour urine samples?
8. How well did your students understand the instructions?
9. Do you think there were any differences in the information provided to you and what was provided to the participating students?
10. We would like to gather some further information about your thoughts regarding sodium intake and health. Could you please indicate on a scale of 1-5 how much you agree or disagree with the statement “In the long term, eating too much salt during childhood may have harmful effects on children’s health”. (1= strongly disagree, disagree ,neither agree nor disagree, agree or 5=strongly agree)
11. Medical research suggests that children eat more salt than is good for them. On a scale of 1-5, do you think more action needs to be taken to reduce the salt in foods targeted at children? (1= strongly disagree, disagree ,neither agree nor disagree, agree or 5=strongly agree)
12. Please share any additional comments/feedback you may have about the study overall, especially if it could help to improve our methods for next time?
